# Model Organism Modifier (MOM): a user-friendly Galaxy workflow to detect modifiers from genome sequencing data using *Caenorhabditis elegans*

**DOI:** 10.1093/g3journal/jkad184

**Published:** 2023-08-16

**Authors:** Tatiana Maroilley, K M Tahsin Hassan Rahit, Afiya Razia Chida, Filip Cotra, Victoria Rodrigues Alves Barbosa, Maja Tarailo-Graovac

**Affiliations:** Department of Biochemistry and Molecular Biology, Cumming School of Medicine, University of Calgary, Calgary, AB T2N 4N1, Canada; Department of Medical Genetics, Alberta Children's Hospital Research Institute, University of Calgary, Calgary, AB T2N 4N1, Canada; Department of Biochemistry and Molecular Biology, Cumming School of Medicine, University of Calgary, Calgary, AB T2N 4N1, Canada; Department of Medical Genetics, Alberta Children's Hospital Research Institute, University of Calgary, Calgary, AB T2N 4N1, Canada; Department of Biochemistry and Molecular Biology, Cumming School of Medicine, University of Calgary, Calgary, AB T2N 4N1, Canada; Department of Medical Genetics, Alberta Children's Hospital Research Institute, University of Calgary, Calgary, AB T2N 4N1, Canada; Department of Biochemistry and Molecular Biology, Cumming School of Medicine, University of Calgary, Calgary, AB T2N 4N1, Canada; Department of Medical Genetics, Alberta Children's Hospital Research Institute, University of Calgary, Calgary, AB T2N 4N1, Canada; Department of Biochemistry and Molecular Biology, Cumming School of Medicine, University of Calgary, Calgary, AB T2N 4N1, Canada; Department of Medical Genetics, Alberta Children's Hospital Research Institute, University of Calgary, Calgary, AB T2N 4N1, Canada; Department of Biochemistry and Molecular Biology, Cumming School of Medicine, University of Calgary, Calgary, AB T2N 4N1, Canada; Department of Medical Genetics, Alberta Children's Hospital Research Institute, University of Calgary, Calgary, AB T2N 4N1, Canada

**Keywords:** *Caenorhabditis elegans*, genetic screening, modifiers, bioinformatics pipeline, short-read whole genome sequencing, galaxy

## Abstract

Genetic modifiers are variants modulating phenotypic outcomes of a primary detrimental variant. They contribute to rare diseases phenotypic variability, but their identification is challenging. Genetic screening with model organisms is a widely used method for demystifying genetic modifiers. Forward genetics screening followed by whole genome sequencing allows the detection of variants throughout the genome but typically produces thousands of candidate variants making the interpretation and prioritization process very time-consuming and tedious. Despite whole genome sequencing is more time and cost-efficient, usage of computational pipelines specific to modifier identification remains a challenge for biological-experiment-focused laboratories doing research with model organisms. To facilitate a broader implementation of whole genome sequencing in genetic screens, we have developed Model Organism Modifier or MOM, a pipeline as a user-friendly Galaxy workflow. Model Organism Modifier analyses raw short-read whole genome sequencing data and implements tailored filtering to provide a Candidate Variant List short enough to be further manually curated. We provide a detailed tutorial to run the Galaxy workflow Model Organism Modifier and guidelines to manually curate the Candidate Variant Lists. We have tested Model Organism Modifier on published and validated *Caenorhabditis elegans* modifiers screening datasets. As whole genome sequencing facilitates high-throughput identification of genetic modifiers in model organisms, Model Organism Modifier provides a user-friendly solution to implement the bioinformatics analysis of the short-read datasets in laboratories without expertise or support in Bioinformatics.

## Introduction

Genetic modifiers are variants that can modify the phenotypic outcome of another variant, such as a primary disease-causing variant. In evolution, genetic modifiers are the result of the development of redundancies between genes and pathways to help overcome detrimental mutations in essential loci. Modifiers are a large source of phenotypic plasticity and their role as genomic buffers for deleterious variants is becoming increasingly prevalent in the literature (Chari and Dworkin 2013; Cooper *et al*. 2013; Kolder *et al*. 2015; van Leeuwen *et al*. 2017). In Mendelian disorders, it has been observed that patients with the same genotype can have variable phenotypes, ranging from no phenotype to severe phenotype (Slavotinek and Biesecker 2003; Cutting 2010; Rahit and Tarailo-Graovac 2020). In addition, pathogenic genotypes have been identified in populations considered “healthy” (Chen *et al*. 2016; Tarailo-Graovac *et al*. 2017). These modifier-modulated variabilities continue to pose a challenge in identification, and hence only a small number of genetic modifiers in Mendelian disorders have been reported (Rahit and Tarailo-Graovac 2020). Despite the advent of Whole Genome Sequencing (WGS) technologies, the identification, interpretation, and functional validation of genetic modifiers remains challenging at every step.

Traditionally, genetics modifiers were explored in humans by genome-wide association studies to link traits with genotypes, or exhaustive computing methods like multifactor dimensionality reduction. But rare diseases limit such studies due to the scarcity of cases. For Mendelian diseases, we have relied on genetic screens in model organisms like roundworms (*Caenorhabditis elegans)* (O’Connell *et al*. 1998), *Drosophila* (Ou *et al*. 2021), and mice (Buchovecky *et al*. 2013).

These model organism-based experiments are usually designed to screen for the modifier variants where the target variant and its detrimental phenotype (i.e. 100% lethality) is known. The modifier variants are screened through forward-mutagenesis techniques that result in random mutations (using mutagens such as ENU and EMS). Strains that present with a different observable phenotype suggest a modifier effect [e.g. reduced embryonic lethality which allows strain propagation despite the presence of the original lethal variant (Geister *et al*. 2018; Wiesenfahrt *et al*. 2018; Jean *et al*. 2021)]. Because mutagens create hundreds of random mutations, backcrossing is often used to eliminate mutations with no observable effect on the phenotype of interest to narrow down the genetic modifier candidate variant. Overall, such a protocol may be laborious and time-consuming.

Advances in high-throughput sequencing technologies have enabled cost-efficient and effective methods for the identification of genetic modifiers in human and model organisms. In previous studies, we and others showed that the use of short-read WGS (srWGS) and bioinformatics analyses, as opposed to the usual back-crossing approach, reduces the cost and time of genetic screens in *C. elegans* (Doitsidou *et al*. 2010; Minevich *et al*. 2012; Joseph *et al*. 2018; Jean *et al*. 2021), increases the ability at exploring more mutated genomes with potential modifier candidates and allows accurate detection of intragenic and extragenic modifiers.

While there exist many bioinformatics tools for various steps of srWGS data analysis, these typically require some fundamental bioinformatics training such as the use of shell commands, specific programming language, or Unix system to efficiently run those. They also require computational resources to handle data, develop, and run workflows for analyses. These limitations along with the lack of personnel with bioinformatics experience in genetics teams can restrict the ability to implement genomics and high-throughput sequencing methods.

Galaxy is a popular open-source web-based bioinformatics platform (The Galaxy Community 2022). It allows its users to run a broad collection of bioinformatics tools via a user-friendly web interface without requiring bioinformatics expertise. For example, an RNA sequencing data analysis pipeline specific to *C. elegans* has been published previously as a Galaxy workflow (Amrit and Ghazi 2017) as well as an organism-agnostic method (Batut *et al*. 2021). However, to this date, there is no published user-friendly pipeline catered for modifier identification.

To help overcome this barrier and facilitate the development of high-throughput genetic screens based on srWGS in model organisms, we have developed a pipeline that was previously used by Jean *et al*. (2021). In the present study, we have implemented this pipeline in Galaxy to make it accessible to everyone. This Galaxy workflow named Model Organism Modifier (MOM) can detect and filter variants in *Caenorhabditis elegans* to generate a Candidate Variant List (CVL) containing potential genetic modifier variants. In addition, we offer here guidelines to manually curate the list of variants created by MOM to prioritize the top modifier candidates.

## Methods

MOM Galaxy workflow has been implemented to run the pipeline developed to detect genetic modifiers in genetic screening in *C. elegans* (Jean *et al*. 2021) in the Galaxy environment (The Galaxy Community 2022), combining various tools alongside preconfigured parameters.

### Data generation and preparation

MOM Galaxy workflow has been designed to analyze paired-end srWGS data. Thus, the main inputs are the two FASTQ files (R1 or forward and R2 or reverse) containing the raw reads from the sequencing machine. The workflow necessitates additional files that are listed in Table 1. Among these files, for analyses of *C. elegans* genomes, we have made available for download the Gene Ontology (GO) terms, Human Orthology, and Exclusion List that can be found at https://github.com/MTG-lab/MOM.

**Table 1.** Input files to run MOM Galaxy workflow.

Galaxy File ID	Type	Function
Forward Fastq File (R1)	FASTQ (.fastq.gz)	Contains forward reads and quality information—raw data from the sequencing machine
Reverse Fastq File (R2)	FASTQ (.fastq.gz)	Contains reverse reads and quality information—raw data from the sequencing machine
Reference genome	FASTA (.fa.gz)	Contains the sequence of the reference genome
Sample ID	TEXT (.txt)	Contains an ID for your sample
GO terms	Tabulated (.tsv)	Contains GO term to annotate the variants list
Human Orthology	Tabulated (.tsv)	Contains information regarding human genes to annotate the variant list
Exclusion List	Tabulated (.tsv)	Contain a list of variants detected in other samples that should not be considered as a candidate

### Generation of the GO terms file

GO terms file has 3 mandatory columns, separated by a tabulation:

1. Gene_ID (as displayed by SnpEff in the VCF file)
2. GO term
3. Definition of GO term

The GO term file that we have provided for MOM has been generated by extracting GO terms for each *C. elegans* gene from the Parasite WormBase webpage (https://parasite.wormbase.org/index.html) using the BioMart tool, with the following parameters:

1. Query Filters: SPECIES > *C. elegans* (PRJNA13758) [WS281]
2. Output Attributes: GENE > Gene stable ID; GENE ONTOLOGY > GO term name AND GO term definition

For each gene, multiple GO terms and definitions were reported. We modified the result of that query to concatenate all GO terms and GO definitions into one line for each gene. This formatting is essential for the processing of the file by MOM and to avoid duplication of variants in the final CVL.

### Generation of the Human Orthology file

The Human Orthology file has 6 mandatory columns:

1. Gene_ID (as displayed by SnpEff in the VCF file)
2. Gene Name
3. Transcript ID
4. Human Gene ID
5. Human Gene Name
6. OMIM-associated disease

The Human Orthology file we have provided through our GitHub repository has been generated by the extraction of appropriate columns for the OrthoList2 study (Kim *et al*. 2018). For some genes, multiple human ortholog genes were reported. We modified our file by concatenating all Human Gene IDs, Human Gene Name, and OMIM (Online Mendelian Inheritance in Man, OMIM, 2023)-associated diseases into only one line for each *C. elegans* gene. This formatting is essential for the processing of the file by MOM and avoids duplication of variants in the final CVL.

### Generation of the Exclusion List file

The Exclusion List file has 4 mandatory columns:

1. Chromosome
2. Position
3. Reference allele
4. Alternate allele
5. Sample (optional)

The example Exclusion List file provided with this paper has been generated by extracting variants detected in *C. elegans*N2 and CB4856 genomes processed in the Tarailo-Graovac group. We recommend the user build their own Exclusion List while working on a genetic screen. The choice of the variants that should be on the Exclusion List would depend on the experiment design. The Exclusion List aids in removing variants that are not relevant because of sequencing errors inherent to each protocol and frequency. For example, one can combine variants detected in other strains, for which genomes have been extracted, sequenced, and aligned through the same protocols. By adding more samples to the Exclusion List, it is to be expected that the number of variants in the final CVLs will be reduced (see Results).

MOM will automatically remove all variants present in the Exclusion List file from the final candidate lists. If you are studying backcrossed genomes, ideally, the samples you are processing, and any parental strain should not be present in your database. Alternatively, you can design your Exclusion List by including all your samples (sample of interest and parental strain) but removing variants according to an appropriate frequency threshold (e.g. only keep variants not present in any strain or one other strain).

The MOM Galaxy workflow can be used to obtain the variants present in the samples to be added to the Exclusion List. For that, the MOM workflow needs to be run first on a strain user wishes to include in the Exclusion List. It will produce the raw VCF file (including all good-quality homozygous variants called by Freebayes) named “*variantcalling_freebayes.vcf*”. By taking the variants from this VCF file according to the Exclusion List file format (tabulated file with the mandatory fields *Chromosome*, *Position*, *Reference allele*, *Alternate allele*), the Exclusion List can be produced. Subsequently, this Exclusion File can be used to re-run the analysis of the mutagenized strains of interest to filter out any variant present in other strains such as the parental strain. To facilitate the creation of the Exclusion List, we have implemented an auxiliary workflow named *Build_Exclusions_List_MOM_workflow_v1* (available at https://usegalaxy.org/u/tmaroilley/w/buildexclusionlistmomworkflowv1) to help the user to build their own Exclusion List (see more details in Supplementary Information and Supplementary Fig. S1).

### Implementation of the MOM workflow

All the tools used in MOM existed in the Galaxy ToolShed (Blankenberg *et al*. 2014) (listed in Table 2). Tools and parameters have been implemented as previously published by Jean *et al*. (2021) for an effective genetic modifier variant detection.

**Table 2.** Tools from Galaxy toolshed used in the MOM workflow.

Tool name	Step	Version	References
FastQC	Read Quality reports	0.11.9	Andrews (2010)
Trimmomatic	Flexible read trimming tool	0.38	Bolger *et al*. (2014)
BWA-MEM	Read mapping	0.7.17	Li (2013)
MarkDuplicates	Flag the duplicated reads	2.18.2.3	“Picard Tools—By Broad Institute”
Freebayes	Variant calling	1.3.1	Garrison and Marth (2012)
Bcftools	Add sample name in VCF header	1.10	Danecek *et al*. (2021)
SnpEff	Variant annotation	4.3.1	Cingolani *et al*. (2012)

**Fig. 1.**
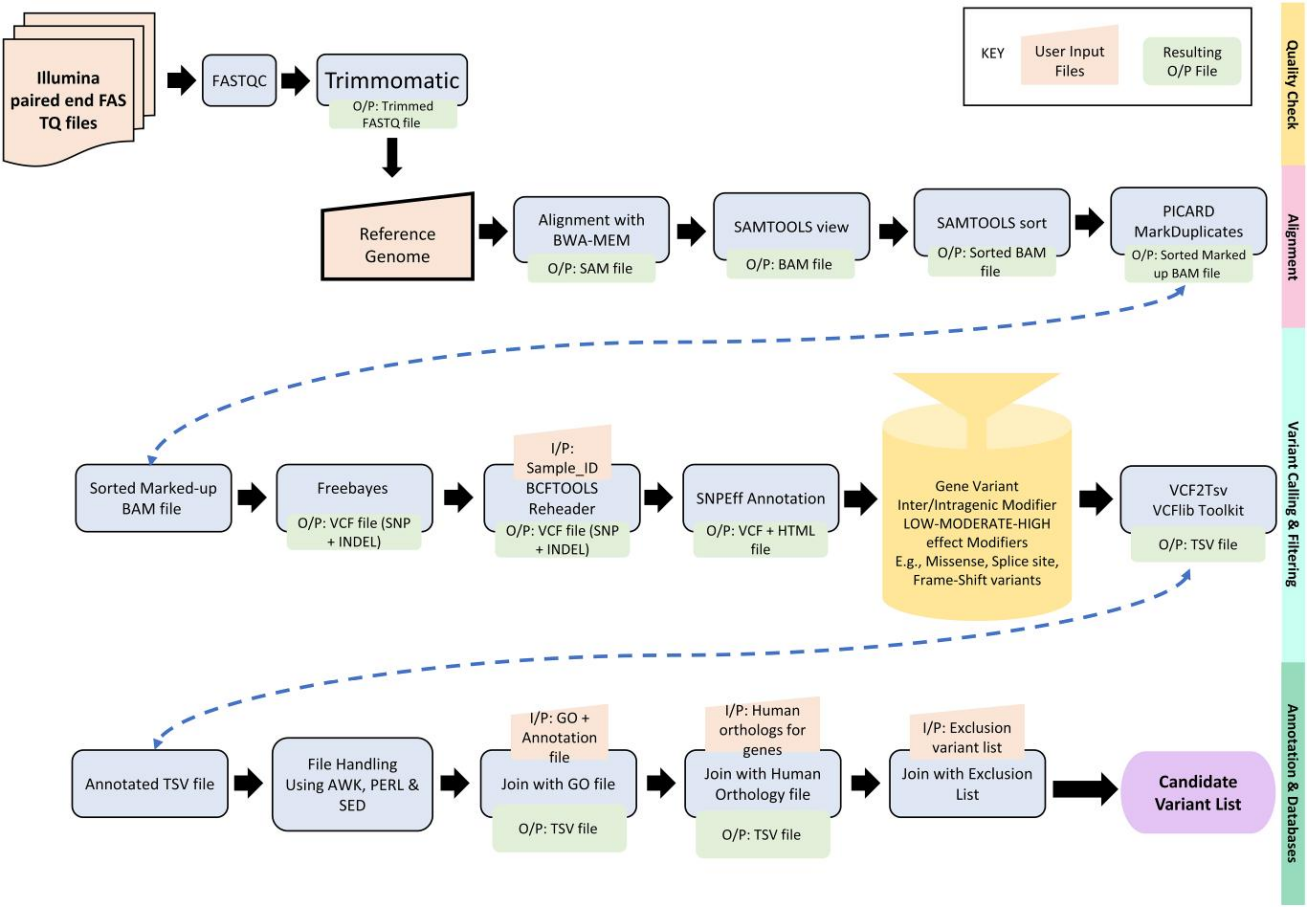
Schematic representation of the main bioinformatics steps implemented in the Galaxy workflow MOM.

The workflow comprises 6 main steps (Fig. 1 and Supplementary Fig. 1) starting with a quality check for the FASTQ files (short-read raw data), trimming the reads, mapping on the reference genome, detecting the variants, annotating the variants, and filtering the variants. All steps are explained in detail below.

### Quality check of the reads with FastQC

FastQC provides quality control of the FASTQ files (R1 and R2). FastQC will create a report (html/web page format) made available in the History (“FastQC on data 1” and “FastQC on data 2”) that will display assessment of various quality statistics such as the number of reads, base quality, read quality, N content, *k*-mer content, read length (for more information, see https://www.bioinformatics.babraham.ac.uk/projects/fastqc/).

### Read trimming and adapter removal with trimmomatic

The next steps consist of removing the adapter sequences (by default TruSeq3 paired-ended, for MiSeq and HiSeq) and trimming low-quality bases at the edges of the reads using Trimmomatic from both FASTQ files (R1 and R2). Trimmomatic will produce 4 FASTQ files as it separates paired and unpaired reads (paired reads are forward read from the R1 FASTQ file for which the mate, or reverse read, is found in the R2 FASTQ file). Paired FASTQ files will be used for downstream analyses. Paired and unpaired FASTQ files are hidden in the History.

### Read alignment with BWA-MEM, marking the duplicates with MarkDuplicates and quality check

The paired reads are then aligned on the reference genome using the software BWAMEM. BWA creates a BAM file (compressed file) containing the position where each read was aligned with additional information such as the quality of the mapping. Reads are sorted by coordinate. We then use MarkDuplicates (Picard Tools) to flag the duplicated reads, resulting from amplification steps during the library preparation, so that such reads would be ignored in downstream analyses. The final BAM file and its index are available in the Galaxy History (“MarkDup_BWA_Trim.bam”) for download to visualize alignments with tools such as the Integrative Genomics Viewer (IGV) (Robinson *et al*. 2011). At the end of this step, we again perform a quality check using FastQC of the BAM file. The FastQC report is available for download and consultation in the Galaxy History (“FastqQC_bamfile”).

### Variant calling with freebayes

Freebayes is a sequence-based variant caller (Garrison and Marth 2012) that uses a haplotype-based Bayesian inference to evaluate the semantic consistency of the sequence. It produces a VCF file with the detected Single Nucleotide Variant (SNV)s and InDels, sorted by coordinates. The VCF file (available in the Galaxy History as “variantcalling_freebayes.vcf”) gives multiple information such as reference and alternate allele, homozygous or heterozygous state based on the number of reads supporting each allele or quality. Freebayes has been implemented in MOM to report by default only: SNPs and InDels (1) in regions covered by at least 5 reads and a maximum of 200 reads with a minimum of alignment quality of 30, (2) with at least 5 reads and 90% of the reads (homozygous) supporting the alternative allele, and (3) with alleles supported by bases with quality over 30. We then use the Bcftools readheader to annotate the VCF file with the Sample ID provided in the *Sample_ID.txt* text file.

### Variant annotation with SnpEff and filtration

SnpEff annotates variants in the VCF by adding information regarding the effect of the variant on genes, if any (Cingolani *et al*. 2012). The variants can be of HIGH, MODERATE, LOW, or MODIFIER impact, depending on if they are missense, frameshift, synonymous, 5′UTR, 3′UTR for instance. For more information regarding variant annotation by SnpEff, see http://pcingola.github.io/SnpEff/se_inputoutput/. By default, SnpEff has been implemented in MOM to only annotate based on canonical transcripts, and not to report further DOWNSTREAM, UPSTREAM, INTERGENIC changes or the 5′UTR, and 3′UTR variants. SnpEff creates an annotated VCF (“*snpeff_variantcalling_freebayes.vcf*”) and a summary statistic file (html/web page) available in the Galaxy History.

To facilitate the identification of candidate modifiers, we have implemented the automated filtration and annotation processes previously used by Jean *et al*. (2021). This filtration is performed by default based on the criteria defined in Table 3. This process results in the creation of a CVL—or CVL—containing potentially disrupting candidate modifiers, among other variants that would need to be manually curated.

**Table 3.** Automatized filtration process.

Step #	Filter on attribute	Filtration criteria
1	Variant type	Filter out intergenic, downstream, upstream, 5′UTR, and 3′UTR variants
2	Variant Impact	Report only variants with HIGH or MODERATE impact on a gene
3	Variant frequency	Filter out variants present in the Exclusion List

In addition to the annotation of the variant performed by SnpEff, our process adds information on the gene: gene function defined with GO terms, the definition of GO terms, human ortholog, and the implication of the human ortholog gene in a rare syndrome reported in OMIM. The final file, called CVL, is a tabulated file that can be downloaded from the Galaxy History (“*candidate_variants_list_filtered_annotated.tsv*”).

### MOM workflow outputs

With the default configuration, the MOM workflow will produce 32 files. All of these are accessible through Galaxy *History*. To declutter the output space, MOM will automatically hide the intermediate file and show the ones that are necessary for downstream analysis. For advanced use-case scenarios or debugging purposes, the intermediate files can be accessed by clicking on the icon “*Include hidden*” on the top of the History (see Fig. 3). For each sample, MOM will create 8 output files that are visible by default (ordered by importance):

1. The first in the list (last created) in the CVL is called “*candidate_variants_list_filtered_annotated.tsv*”. The format of the CVL is described in the next section and a screenshot is available in Supplementary Fig. 2.
2. Two VCF files are available: “*variantcalling_freebayes.vcf*” and “*snpeff_variantcalling_freebayes.vcf*”. The first is the raw VCF produced by Freebayes containing all good-quality homozygous variants detected in the sample. The second is the same list of variants after annotation with SnpEff.
3. The bam file containing reads aligned along the reference genome (after trimming and marking the duplicates) is available under the name “*MarkDup_BWA_trim.bam*”.
4. An html page providing statistics on the annotated variants is available to the user: “*snpeff_variantcalling_freebayes*”.
5. Three other html pages are available to the user reporting quality statistics of the fastq files and the bam files, namely: “FASTQ on data [number]: Webpage” and “*FastQC_bamfile*”.

### CVL description

The CVL describes 22 attributes (columns) for each reported variant (row). These attributes are described in Table 4. Supplementary Fig. 2 is a screenshot of a CVL provided as an example.

**Table 4.** CVL entries.

Entry	Name	Description
1	Chr	Chromosome where is located the variant
2	Position	Genomic position of the variant
3	Reference	Reference allele at the variant position
4	Alternate	Variant allele
5	WormBaseID	WormBase gene ID
6	GeneID	Gene Name
7	Transcript	Transcript ID
8	Type	Type of variant: snp, del (small deletion), ins (small insertion, including small duplication)
9	Effect	Effect of the variant on the gene sequence (frameshift, missense, splicing variant, etc.)
10	Impact	Predicted level of disruption (HIGH, MODERATE, LOW)
11	Location	Protein coding region or not
12	Change	Nucleotide change
13	AAchange	Amino acid change
14	Exon	Affected exon
15	Depth	Number of reads covering the genomic position
16	AlternateReads	Number of reads supporting the variant (all of them in case of homozygous variant)
17	Gene_Definition	Gene definition
18	GOterms	GO terms reported for the affected gene
19	GOdescription	Description of the different GO terms
20	EnsemblID_HumanGene	Ensembl ID for human ortholog genes
21	HumanGeneID	Gene Name for human ortholog genes
22	OMIMdisease	Rare diseases reported associated with the gene in humans (source: OMIM)

### Evaluation of the performances of the MOM Galaxy workflow

To assess the MOM Galaxy Workflow, we ran MOM on the strains published by Jean *et al*. (2021) and Joseph *et al*. (2018). For Jean *et al*. (2021), the dataset was downloaded from the NCBI BioProject repository PRJNA761686. For Joseph *et al*. (2018), the dataset was downloaded from the NCBI BioProject repository PRJNA415825. In addition, as variants in Joseph *et al*. (2018) were reported according to the ce10 *C. elegans* reference genome, we used The Lift Genome Annotations tool (UCSC) to transform the genomic positions from ce10 to ce11. For both datasets, we ran 2 tests: one with an Exclusion List containing only variants detected in N2 and CB4856 strains, and one with an Exclusion List containing additional genomes from the screening. To obtain the second Exclusion List for each screen, we first ran MOM of each available sample, then extracted the raw vcf (variantcalling_freebayes.vcf), and pulled together the calls in the Extension List format.

## Results

### How to run MOM

The process of running MOM can be outlined in 3 major steps: (1) loading/importing the workflow, (2) uploading the data and supporting files, and (3) running the workflow. In the following sections, we describe this process. Then we explain how to obtain the results and interpret them.

### Requirements to run MOM Galaxy workflow

Galaxy is a user-friendly web interface to run bioinformatics analyses (The Galaxy Community 2022). To run the MOM Galaxy workflow, it is necessary to have the latest version of a web browser such as Google Chrome, Mozilla Firefox, or Microsoft Edge and to create an account on Galaxy (https://usegalaxy.org/login).

### Loading MOM workflow

The pipeline is available as a publicly shared workflow on the main Galaxy server (http://usegalaxy.org) at https://usegalaxy.org/u/tmaroilley/w/momv1. Users can also find the workflow with the name “*MOM.v1”* under the author names “*tmaroilley”*. To use this workflow on the Main Galaxy server, click on the *Shared Data* tab on the top menu bar and then select *Workflows*. Search for MOM and select the workflow with the name “*MOM.v1”* under the author name “*tmaroilley”*. To import the MOM workflow to your account, click on *Import Workflow*, the icon on the right top corner.

Alternatively, the pipeline is also available on our GitHub repository (https://github.com/MTG-Lab/MOM) for import and use on any Galaxy server. To import this downloadable workflow, click on the *Workflow* tab on the top menu bar and then select *Import*. The user can directly enter the URL of the workflow file (*.ga* file) from the GitHub repository in the *Archived Workflow URL* field to avoid downloading the file. In case the user wants to upload the file, it is possible to do so by clicking on the *Browse* button of the *Archived Workflow File* field.

Once imported, the workflow will appear on the user's workflow list which can be viewed by clicking on *Workflow* tab on the top menu bar. The user can click on the play button on the right side of the workflow entry to run the pipeline.

### Uploading dataset in Galaxy

**Fig. 2.**
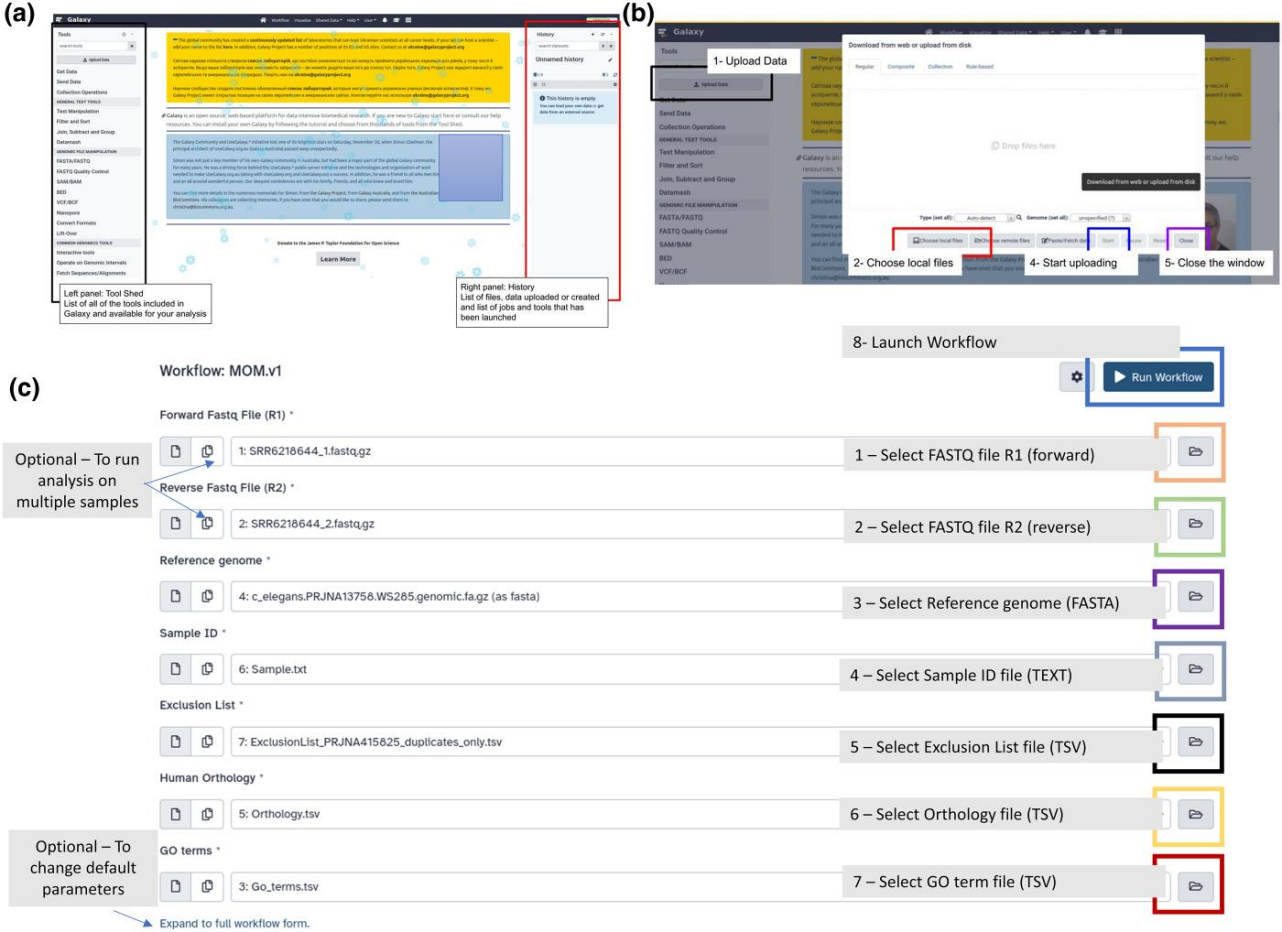
Overview of Galaxy and the workflow webpage. (a) Annotated screenshot of the main page on Galaxy showing on the left the toolshed, and on the right side, the History—displaying jobs status and files. (b) Annotated screenshot of the window allowing uploading files into a History on Galaxy. (c) Annotated screenshot of the Galaxy Workflow MOM main page displaying all necessary input files.

Before running the workflow, input datasets should be uploaded to the Galaxy history (right panel of the main page in Galaxy—Fig. 2a). For a list of input files, see Table 1. To upload a file in Galaxy history, follow the steps (see Fig. 2b):

1. Click on “Upload Data” (Upper left corner)
2. Click on “Choose local files” in the pop-up window
3. Select the files you want to upload on Galaxy from your computer
4. Start uploading by clicking on “Start”
5. Close the window by clicking on “Close”

After successful upload, the input files will appear on the current history. We recommend always running the MOM workflow on a specific sample in a new History to avoid conflict and confusion with output files produced by previous analyses.

### Running MOM on Galaxy

The Run MOM Workflow page is composed of a form (see Fig. 2c) where the user can select the file for each input necessary to run the MOM workflow (see Table 1 for the list of inputs). Once all input files are added, the user can click on the *Run Workflow* button (upper right corner—see Fig. 2c) and the MOM workflow will be automatically invoked in the History. Of note, once launched, the different jobs that are part of the workflow will run once resources on the Galaxy server are available and/or previous steps have successfully worked. The user may close the tab or browser or shut down the computer as this will not impact the processing in any way as the processing is running remotely on the cloud and the results will be available once the job is done.

**Fig. 3.**
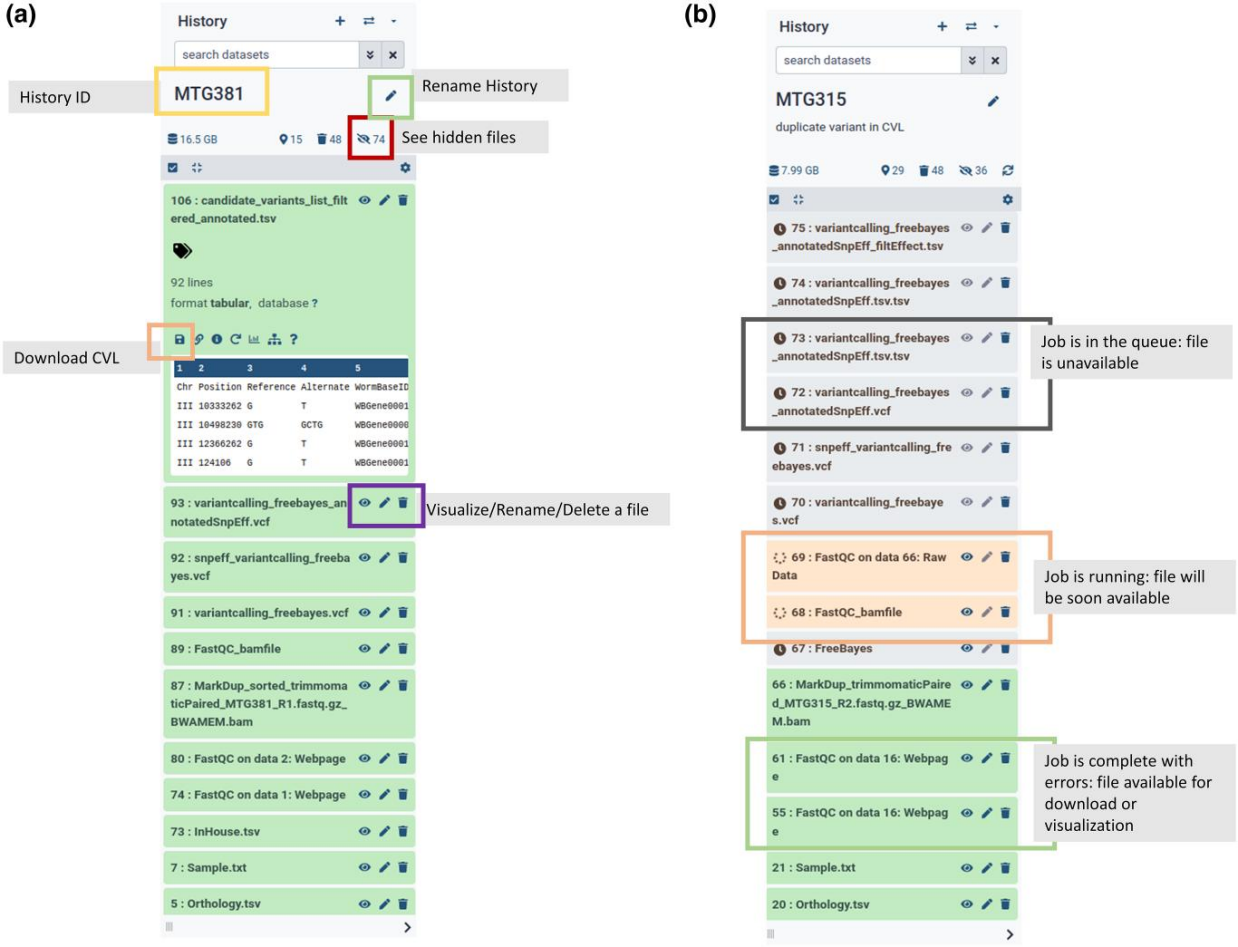
Overview of the Galaxy history. (a) Annotated screenshot of a History in Galaxy showing the different settings available to the user. (b) Annotated screenshot of a History in Galaxy showing some of the different statuses that a job can display: pending, running, done.

The color of the files will tell the status of the run. Gray-colored files with a clock symbol are waiting to be processed, orange color codes with a rotating circle represent the files that are currently running, green ones with no symbol before the name are successfully finished and generated, and red-colored steps with a cross failed to run (Fig. 3a). Users can check the error message to solve the issue.

Once the processing starts, the user will be able to see the job processing page. For a *C. elegans* genome, the whole process takes on average 2.5 hours to run on 30X srWGS data, considering zero wait-time for Galaxy resources (the immediate availability of resources on Galaxy).

Upon successful completion of the job, the output CVL can be found in Galaxy History. Alongside the output CVL, the pipeline also generates seven other output files (see Methods). Outputs from most tools of the pipeline are under the hidden section in the History to declutter the main History and increase the readability of the output window. It is possible to access these hidden output files by enabling the “show hidden” option in the History (Fig. 3b).

By default, MOM Galaxy Workflow will analyze one sample at a time. Galaxy offers an option to run a Workflow on several samples by automatically creating different “History” (see Supplementary Information and Supplementary Fig. 3).

### Download alignment files and CVLs

To download the files, use the History on the right of the main window. Under each available file, the left icon can be used to download the file (see Fig. 3). When you download the alignment files (BAM file), it is important to also download the index files, and store that file in the same folder as the alignment files. The index files are used by many tools to access genomic data more efficiently.

### Manual curation

The first step in the manual curation is the visualization of the variants reported in the CVL. Multiple tools have been published over the last decade to visualize sequencing data. The IGV (https://software.broadinstitute.org/software/igv/) (Robinson *et al*. 2011) is one of the most popular, and here, we will explain its usage for further filtering of the candidate variants reported by MOM.

**Fig. 4.**
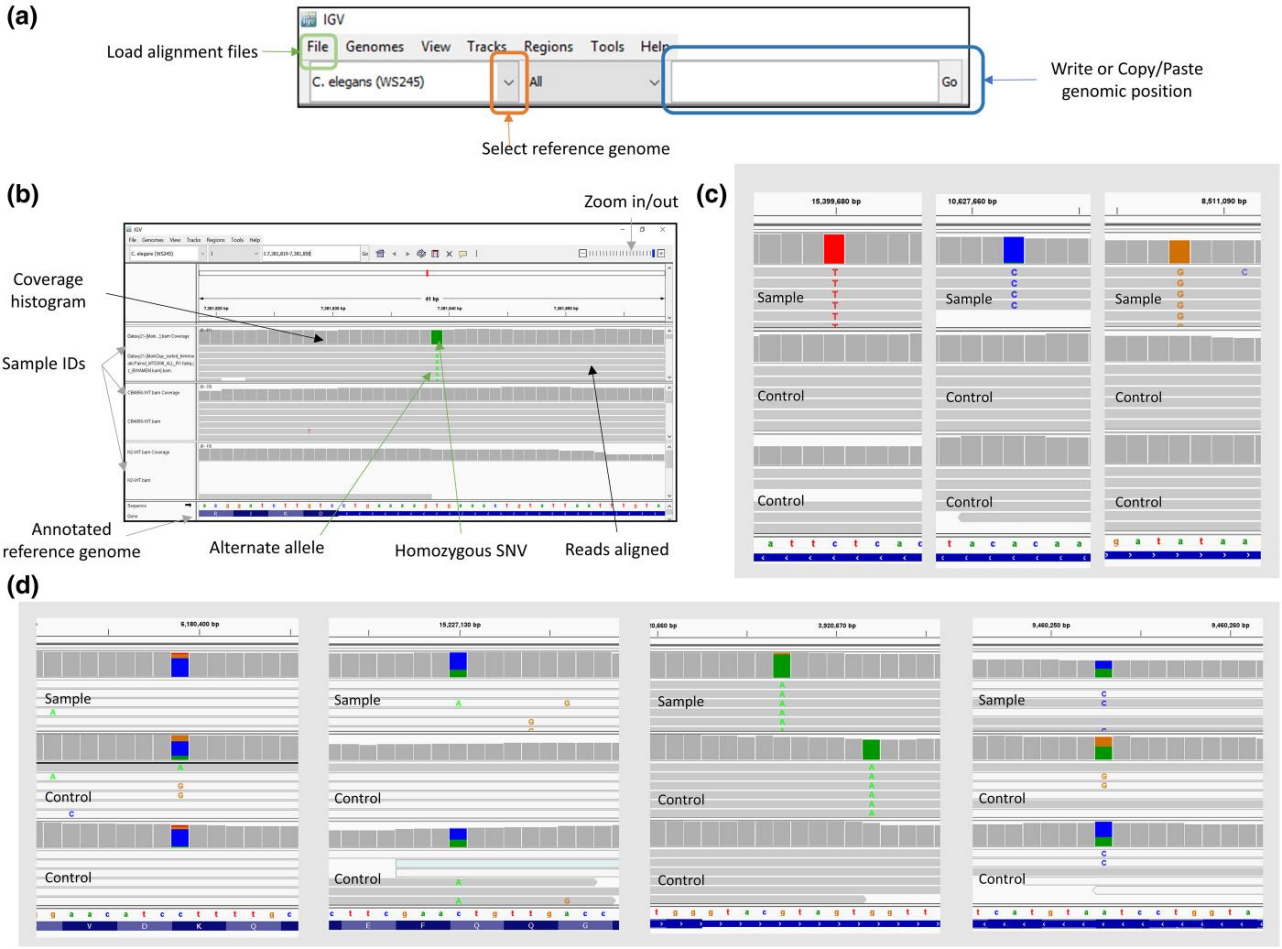
Overview of the IGV and how to use it for manual curation through visualization. (a) Annotated screenshot of the Search bar on IGV. (b) Annotated screenshot of the tracks as displayed on IGV when visualizing WGS datasets. (c) Screenshots of homozygous SNVs of good-quality as visualized on IGV. (d) Screenshots of SNVs of bad quality as visualized on IGV.

To start, download IGV at https://www.broadinstitute.org/software/igv/download. On the main window of IGV, you might need to change the Reference Genome. Click on the drop-down menu in the top left corner to change the reference genome to the species you are analyzing (Fig. 4a). Then upload the alignment files or bam files (previously downloaded from your Galaxy history—see Step *Uploading dataset in Galaxy*). Note that IGV needs index files that can be downloaded along with the bam files from your Galaxy history. The indexes must be stored in the same folder as the alignment files. To load your alignment files, click on “File” in the Menu bar on the top left corner of the main IGV window. Choose “Load from file” from the drop-down menu and select your bam files. Note that IGV allows you to open several alignment files at the same time, facilitating comparison between samples. We recommend loading your sample of interest along with at least one control (such as the N2 strain in *C. elegans*). To ensure efficient visualization, we recommend not loading more than five different samples at the same time.

The key features of the visualization of aligned read in IGV are described in Fig. 4b. At the bottom of the main window, the reference genome is displayed (gene structures and sequences). In brief, samples will be displayed in two tracks. The upper track represents a histogram of the coverage (the number of reads aligned at this position). The lower track is the visualization of the reads themselves. They are represented by arrows (in different directions for paired-end libraries, depending on if they are forward or reverse reads). By default, IGV colors them gray. If a certain region/position displays specific characteristics, they will be colored differently. When a read carries a base different from the reference sequence, it is written on the read. In addition, when the alternate allele is A, bases are colored in green, T in red, G in orange, and C in blue. When several reads are carrying an alternative allele, the histogram on the top track will display a color (same base color code), correlated with the percentage of those reads compared to the coverage.

To visually assess variants (e.g. any interesting variant from CVL), write or copy/paste the genomic location of a variant in the text box in the middle on the top menu bar and click on “Go” (or press Enter on your keyboard). Examples of possible true and false positive SNVs visualized on IGV are displayed in Fig. 4c and d.

In the next section, we are describing characteristic signatures of SNVs that have a high probability of being a true variant, based on sequencing data, and variants that might be false positive due to sequencing, alignment, or detection errors. Those criteria have been tailored for genetic screens aiming to uncover homozygous genetic modifiers created by a mutagenesis process such as EMS or ENU. Such criteria must be revised depending on the study and the goal.

Here are the characteristics of a “good” SNV (with a high probability of being true and experimentally validated) based on genomic data:

1. Homozygous variant should show mostly reads carrying the alternative allele (written directly on the read) and the histogram on the upper track should show only one color; while heterozygous should show 50% of reads with the reference base and 50% of reads with the alternate allele and the histogram should have 2 colors.
2. Coverage of at least 5 reads.
3. At least 5 reads support the alternative allele.
4. Maximum 2 alleles are observable.

Here are the characteristics of a variant that we recommend discarding:

1. The coverage is low in the region—there is a high risk that the allele observed here is a sequencing error.
2. The region is covered by white reads (IGV code for reads that can align on multiple locations)—suggesting a repetitive region.
3. The variant is present in controls (other strains like N2)—as we are looking for a genetic modifier created by random mutagenesis and able to modify the outcome of a primary variant, it is unlikely that this variant can be seen in controls (polymorphism).
4. There are other variants in its vicinity—which could suggest alignment errors.
5. More than two different alleles can be observed at the same position—which could suggest sequencing or alignment error.
6. The SNV is at the boundary of a clear disruption of the sequence (e.g. drastic reduction in coverage suggesting a deletion or increase in coverage suggesting a duplication).

### Prioritizing modifier variant

Most of the modifier prioritization techniques involve manual functional analysis of each variant (Slavotinek and Biesecker 2003; Cutting 2010; Rahit and Tarailo-Graovac 2020). After manual curation of the CVL by visualization of the alignment data, here are the characteristics that can help prioritize candidate modifiers:

1. Variant with a HIGH impact on a gene.
2. Variant affecting a gene with similar functions in pathways as a gene of interest based on GO terms and gene definition (extragenic modifier) or variant affecting the gene of interest (intragenic modifier).
3. Variant affecting a gene with a human ortholog gene associated with a disease with consistent features to the gene of interest.

If no compelling candidate with HIGH impact is identified, the next variants to be considered are (1) MODERATE impact variants, (2) SYNONYMOUS variants, and (3) 5′UTR and 3′UTR variants. If at these stages no candidate modifiers have been identified, one should focus on larger events: first InDels (included in the MOM CVL) and then structural variants (not included), that could have occurred naturally.

Further publicly available resources can be used to expand the manual curation process behind the information directly available in the CVL. For instance, Wormbase (Davis *et al*. 2022) catalogs functional descriptions of all known *C. elegans* genes in different forms and it can be used to compare the functionality of each candidate variant (potentially modifier) with the query gene (target of modifier).

Primarily, the following sections can help to understand the functional impact of the variant:

1. Gene Ontology: GO represents gene functionality in the form of a knowledge graph. Wormbase provides GO ribbon and graph. Ribbon gives an overview of the annotated terms for the gene. It shows which term is more generalized and which terms are specific. For comparison, we primarily use the ontology graph. GO consists of three distinct graphs—Biological Process, Cellular Component, and Molecular Function. Every node of GO is functionality, and every edge describes the relationship between 2 functionalities. Generally, child nodes denote more specific functionality while parent nodes denote generalized. By looking at the network and its node, we can understand all the biological, cellular, and molecular functions of the gene associated with it.
2. Phenotypes: This section shows the Worm Phenotype Ontology (WPO). While GO describes functional consequences, WPO shows the phenotypic outcome associated with the gene. WPO also has all the properties of an ontology.
3. Interactions: The interaction module shows the known interactions for the gene. By navigating to other genes and their functionalities we can know about how the gene can modulate other genes. This section has filtration for positive/negative regulatory, suppressing/enhancing interactions, etc.

The current knowledge about a gene's functional consequence and impact on other genes can be summarized using GO, WPO, and interaction. With this information, the candidate gene's function can be related to the gene of interest.

Modifiers are shown to be genetic background dependent. A variant that acts as a modifier in one genetic background (carrying a set of variants) might not act as a modifier in a different background (having a different set of variants). Therefore, it is important to consider all the variants’ functional dynamics to find out the potential modifier variant.

During modifier variant selection, it is common to ignore the alleles of large genes (e.g. *ttn-1*) as they are prone to frequent mutations, especially in an unbiased mutagenized genome.

### Assessment of MOM Galaxy workflow

To validate the biological relevance of results from the MOM workflow, we analyzed 2 sets of data: a set of 14 mutant strains selected in a genetic screen performed on the *zyg-1* gene (Jean *et al*. 2021) and a set of 4 mutants from Joseph and colleagues (Joseph *et al*. 2018). In both cases, we evaluated MOM performance using two different Exclusion Lists: (1) one containing only variants from N2 and CB4856, and (2) one containing parental strain (with no modifier variant) and another modifier (suppressor) mutants. Of note, the main difference between the Joseph *et al*. dataset and the Jean *et al*. dataset is that the mutant strains in Joseph the al. were backcrossed multiple times, reducing drastically the number of variants when compared to the parental strain (before mutagenesis), reducing then the number of modifier candidates to be analyzed. In Jean *et al.*, the sequenced mutant strains were not-backcrossed.

Our assessment shows that the filtration process implemented in the Galaxy Workflow MOM with default parameters allows us to reduce the variant lists from around 2,000 homozygous variants on average to about 100–200 variants per not-backcrossed modifier strain (see Table 5).

**Table 5.** Assessment of MOM to report modifiers in the CVL with an Exclusion List including variants from additional suppressor mutants and parental strains or with an Exclusion List only including variants in N2 and CB4856.

Strain	Confirmed modifier (ce11)	Type	Parameters	Variants	HIGH	MODERATE	LOW	CVL (only N2 and CB4856)	CVL	Confirmed modifier
MTG57	II:5,651,146-C-T	Missense	Default	1939	52	157	71	173 (142;31)	135 (104;31)	YES
MTG192	II:5,650,939-C-A	Missense	Default	1916	51	121	54	136 (104;32)	97 (65;32)	YES
MTG192	II:5,650,947-T-G	Missense	Default	1916	51	121	54	136 (104;32)	97 (65;32)	YES
MTG308	II:5,651,254-G-A	Missense	Default	2444	59	224	90	245 (216;29)	202 (173;29)	YES
MTG309	II:5,651,146-C-T	Missense	Default	2398	54	214	93	231 (198;33)	188 (155;33)	YES
MTG315	II:5,649,756-G-A	5’UTR	Including 5′ and 3′UTR	2368	55	204	93	262 (226;36)	216 (180;36)	YES
MTG320	II:5,651,219-T-C	Synonymous	Including synonymous	1946	48	127	48	140 (105;35)	113 (78;35)	YES
MTG320	II:5,651,252-T-A	Missense	Default	1946	48	127	48	140 (105;35)	100 (65;35)	YES
MTG329	II:5,653,611-A-G	Missense	Default	2059	46	133	68	144 (115;29)	103 (74;29)	YES
MTG354	II:5,650,847-A-C	Missense	Default	1975	44	127	61	137 (108;29)	96 (66;29)	YES
MTG355	II:5,651,254-G-A	Missense	Default	2039	54	125	63	144 (108;34)	101 (67;34)	YES
MTG398	II:5,649,770-T-A	5′UTR	Including 5′ and 3′UTR	1703	39	91	51	120 (86;34)	83 (49;34)	YES
MTG406	II:5,651,146-C-T	Missense	Default	1737	41	150	73	160 (136;24)	127 (103;24)	YES
MTG423	II:5,651,073-G-A	Missense	Default	1925	55	153	68	174 (146;28)	137 (109;28)	YES
MTG426	II:5,650,948-C-T	Missense	Default	1957	40	158	70	173 (142;31)	133 (102;31)	YES
MTG437	II:5,650,846-G-A	Missense	Default	2059	48	157	64	169 (136;33)	131 (98;33)	YES
WY1217	X:17,191,818-5bp-DEL	-	Default	1737	44	97	44	114 (88;26)	3 (2, 1)	YES
WY1211	III:5,452,540-G-A	-	Default	1676	45	111	47	130 (102;28)	6 (6;0)	YES
WY1208	X:13,692,221-G-A	-	Default	1714	49	99	51	119 (90;29)	8 (7;1)	YES
WY1209	II:1,320,065-C-T	-	Default	1813	49	109	52	131 (100;31)	3 (3;0)	YES

Our tests showed that MOM always reported the confirmed modifier variant in the final CVL. On average, 2.5% of the homozygous variants are predicted to have a HIGH impact and 6.8% to have a MODERATE impact on a gene. Then, about 90% of variants are filtered out based on the predicted effect of genes, with on average 178 HIGH/MODERATE impact variants (range 141–268).

For both datasets, we have tested MOM with two different Exclusion Lists: (1) only contains variants from the reference strains N2 and CB4856; (2) for Jean *et al*., contains a dozen strains produced in the *zyg-1* mutagen process (unpublished data) and the parental strain OC14 and for Joseph *et al*., contains all 10 strains made available by the authors (5 pairs of sibling strains, see Supplementary Table 1 in Text S1).

The use of the Exclusion List helps remove even more variants that can be called in reference strains such as N2 or CB4856, but also variants that are shared with other mutants from the same screen and parental strain (before mutagenesis), potentially being recurring sequencing, detection errors or polymorphisms. By using the Exclusion List (1) (only N2 and CB4856), about 18% of variants are further filtered out. While by using a larger Exclusion List (2) on non-backcrossed mutants, about 37% of variants are further filtered out. As a result, the CVL obtained while using a larger Exclusion List will be shortened and more specific, and the manual curation will be less time-consuming.

For specific cases of the Jean *et al*. dataset, for which the modifier variant was a 5′UTR (e.g. II:5,651,146-T-C or II:5,649,756-G-A) or a synonymous variant (e.g. II:5,651,219-T-C), by adjusting the parameters to allow the inclusion of such variants in the CVL, the Galaxy Workflow MOM was able to report the modifier variant in the CVL. For each mutant tested, the final CVL always contained the modifier candidate, previously confirmed experimentally.

## Discussion

The implementation and usage of pipelines for high-throughput sequencing datasets consist of multiple tools and require computational expertise. However, we have shown that srWGS constitutes a time- and cost-effective alternative to backcrossing allowing high-throughput genetic modifier screening in model organisms such as *C. elegans* (Jean *et al*. 2021). To democratize such an approach, we have implemented a pipeline developed by our group and used in Jean *et al*. (2021) on the Galaxy platform. Galaxy Workflows indeed make it easy for anyone not having the computational background to run a pipeline. Thus, we present here MOM, a Model Organism Modifiers Galaxy Workflow for srWGS data processing catered to help in identifying genetic modifiers in model organisms. MOM has been designed to handle automatically and at once the reads from the FASTQ files, to provide a reduced list of potential candidate modifiers. In addition, in this paper, we are presenting the manual process, including visualization of the variants and analysis of the functional impact, to curate this list and identify candidate modifier variants.

Previous software and workflows attempted to democratize the use of WGS among the model organism community. Doitsidou *et al*. (2010) implemented in 2010 a software analysis tool called MAQGene for mutant sequence analysis, but it is no longer supported. Minevich *et al*. (2012) published in 2012 a Galaxy workflow called CloudMap for rapid analysis of mutants from large-scale genetic screens aiming to find the region of the genome that is linked to the phenotype-causing mutation and identify the causal variant. CloudMap requires the mutant to be crossed to a mapping strain (such as CB4856) while MOM has been designed to identify genetic modifiers without the need of crossing. Published ten years ago, the CloudMap workflow now suffers issues at execution due to missing tools in the Galaxy ToolShed. Joseph et al. (2018) have proposed a method to reduce the number of candidate variants called the Sibling Subtraction Method (SSM) by eliminating variants present in both mutant and nonmutant siblings, a similar approach to our Exclusion List.

There are several obstacles to the wide implementation of high-throughput genetic screens. First, the cost of WGS could be prohibitive, but as shown by Jean *et al*. (2021), the drastic reduction of sequencing cost over the past few years is making this approach nowadays cost-effective, especially by the gain of time and labor inherent to other methods such as backcrossing. Second, the involvement of sequencing technologies brings about the necessity of downstream bioinformatic analyses which can refrain groups lacking that type of skillsets. However, Galaxy, among others, is a user-friendly platform that allows nonexpert users to run high-level bioinformatics analyses if implemented and made public beforehand by experts in Bioinformatics. Thus, one of the main goals of MOM, achieved by its implementation on Galaxy, is to allow anyone to run a complete analysis of srWGS datasets as part of a high-throughput modifier genetic screen in model organisms. Third, by allowing the exploration of the entire genomes, the srWGS approach subsequently creates a large amount of data, overwhelming for manual curation without automatized preprocessing of the variant list. Then, in the Galaxy Workflow MOM, not only have we implemented the processing of the short reads (quality check, trimming, alignment, and variant calling), but also, we have implemented the semi-automated filtering process developed in our group and presented in Jean *et al*. (2021). It reduces the list of variants by excluding unlikely candidates and keeps the list manageable size, thereby increasing the chance to identify true modifier variants by manual curation. Finally, the prioritization of variants is challenging because of the lack of standardized techniques for modifier prioritization. We have made available in this manuscript the manual process developed, tested, and assessed in our group based on the visualization of the variants to eliminate the remaining sequencing errors or highly frequent variants missed by MOM, but also on the manual meta-analysis of functional information from various sources to uncover the best candidate modifiers for the gene of interest.

The Galaxy Workflow MOM has been designed to report by default homozygous high-confidence SNVs and InDels with HIGH or MODERATE impact on a gene and unique to the strain analyzed. We have however described in this manuscript how to also include synonymous variants, 5′UTR, 3′UTR, and heterozygous variants. Each change in the parameters will change the final CVL produced by MOM. Through its implementation on Galaxy, each parameter is accessible to the users to adjust it to the specific needs of their current project. The exploration of any other variant type (beyond SNVs and InDels, heterozygous or homozygous, with HIGH or MODERATE impact or synonymous and 5′ and 3′UTR) would require further changes in the parameters and potential adjustments of the bioinformatics filtering process.

The provided pipeline and its parameters are optimized for routine modifier analysis from WGS data of mutagenized genomes. Our solution attempts to cover the most common scenarios in modifier identifications. Although originally developed, tested, and published for *C. elegans* high-throughput genetic screens, by adjusting the input files such as the reference genome, the GO terms, the Orthology, the Exclusion List, and changing the annotation reference used by default in SnpEff, one can apply the Galaxy Workflow MOM to genetic screen implemented in another model organism. In case the project requires more customization or sophisticated data integration, developing a technical pipeline using solutions like Snakemake or NextFlow could be an efficient choice (Garcia *et al*. 2020; Kuzniar *et al*. 2020; Atxaerandio-Landa *et al*. 2022; Petereit 2022). As processing WGS involves the integration of multiple tools, these workflow orchestration frameworks provide a systematic way to integrate different tools without writing customized integration scripts on an ad-hoc basis.

A pipeline carries the limitations of the tools it implements. As such, MOM, by implementing SnpEff to annotate the variants, is limited to interpreting variants in the haplotype. Indeed, SnpEff is not haplotype aware, and some variants could be incorrectly predicted to have a LOW impact. In the future, additional annotation tools could be added. To circumvent that limitation, one could choose to include the variants classified as LOW, when no convincing candidates with predicted HIGH or MODERATE impact were reported in the CVL (see Supplementary Information).

There exist multiple public workflows on Galaxy (Wee and Yap 2021, public workflows on https://usegalaxy.org/workflows/list_published) but ours presents a novel user-friendly pipeline to help identify genetic modifiers from srWGS data in model organisms optimized for generating a short CVL for genetic modifiers. With the Galaxy Workflow MOM, running the pipeline and getting the CVL is straightforward. One limitation of having implemented our workflow on Galaxy is that Galaxy, as an online public platform, can suffer from potential instability, service interruption, or delay in running analyses when the platform is overloaded. In addition, challenges remain in the identification of potential modifier variant candidates. It requires manual evaluation of almost every variant in the list, bottlenecking the throughput of the modifier identification. To the best of our knowledge, there is no published tool dedicated to identifying by prioritization modifier variants from a CVL. It is possible that the modifier variants are highly genetic background dependent and heterogeneous making it a challenge for developing a tool for it. Although machine learning could be a way to approach the problem, it is constrained by the availability of proper datasets. As a solution, we propose guidelines to help the manual curation process and to identify valid genetic modifier candidates. However, experimental validation should always be conducted, by CRISPR (Clustered Regularly Interspaced Short Palindromic Repeats) approaches for instance as proposed by Jean *et al*. (2021).

## Conclusions

Here, we offer a semi-automatized bioinformatic process to allow the implementation of high-throughput genetic screening in *C. elegans* and other model organisms. Indeed, we provide a user-friendly tool to analyze srWGS datasets with state-of-the-art bioinformatics protocols and provide a concise list of potential candidate modifiers or CVL. In addition, we propose detailed guidelines for the manual curation of the CVL and identification of candidate modifier variants before experimental validation.

## Supplementary Material

jkad184_Supplementary_Data

## Data Availability

The Galaxy Workflow pipeline is available as a publicly shared workflow on the main Galaxy server (http://usegalaxy.org) at https://usegalaxy.org/u/tmaroilley/w/momv1. The Exclusion List pipeline is available at https://usegalaxy.org/u/tmaroilley/w/buildexclusionlistmomworkflowv1. The srWGS datasets used to test the Galaxy Workflow MOM are available as described in Jean *et al*. 2021 and in Joseph *et al*. 2018. The database files made available by the authors to run the Galaxy Workflow MOM (GO terms, Human Orthology, and Exclusion List) are available at https://github.com/MTG-lab/MOM. Supplemental material available at G3 online.
