# Supplementary material for: Model Organism Modifier (MOM): a user-friendly Galaxy workflow to detect modifiers from genome sequencing data using Caenorhabditis elegans
Source: G3 (Bethesda). 2023 Aug 16;13(11):jkad184. doi: 10.1093/g3journal/jkad184 (PMC10627290; doi:10.1093/g3journal/jkad184)
Supplement: jkad184_Supplementary_Data [file jkad184_supplementary_data.zip › Supplemental_Figure_Legends_G3-2023-404449.docx]

**Figure S1:** The Galaxy Workflow MOM as displayed on the Galaxy Workflow module, showing the dependencies between the different steps of the workflow.

**Figure S2:** Screenshot of an example of a CVL as the main output of the MOM Galaxy Workflow.

**Figure S3:** Annotated screenshot of the Galaxy Workflow launch page when the user wishes to run several samples at the same time.

**Figure S4**: Screenshots of the Galaxy History showing the main steps to create a VCF Data Collection to be used in the Build_Exclusion_List workflow.
