# Supplementary material for: Model Organism Modifier (MOM): a user-friendly Galaxy workflow to detect modifiers from genome sequencing data using Caenorhabditis elegans
Source: G3 (Bethesda). 2023 Aug 16;13(11):jkad184. doi: 10.1093/g3journal/jkad184 (PMC10627290; doi:10.1093/g3journal/jkad184)
Supplement: jkad184_Supplementary_Data [file jkad184_supplementary_data.zip › Text_S1_G3-2023-404449.docx]

MOM: A user-friendly Galaxy workflow to detect modifiers from genome sequencing data using *C. elegans*

Supplementary Information

Tatiana Maroilley*, K M Tahsin Hassan Rahit*, Afiya Chida, Filip Cotra, Victoria Rodrigues Alves Barbosa, Maja Tarailo-Graovac

*equal first authors

**Keywords**: *C. elegans*, genetic screening, modifiers, bioinformatics pipeline, short-read whole genome sequencing, Galaxy

## Going further with MOM workflow

### Changing Parameters

Before starting the process, one can change the configuration for any tool and step included in the MOM Galaxy Workflow. To do so, click on the “Expand to full workflow form” at the bottom of the input page (See Figure 1). Select the input files only after clicking on the “Expand to full workflow form” as it might reset your inputs.

To expand the parameters on a specific tool, click on “Expand”
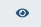
for a step of interest. Once expanded, the tool-wise configuration will be visible, and any parameter can be changed by clicking on the edit icon to appear on the left of each parameter.

### Including synonymous variants

To include SYNONYMOUS variants, before launching the workflow, click on “Expand to full workflow form” (See Figure 1C) and scroll down to “Step 20: Search in text files”. At this stage, we use a regular expression to filter out all variants with no HIGH or MODERATE effect, including synonymous variants (based on SnpEff variant characterization - <http://pcingola.github.io/SnpEff/se_inputoutput/>). Change “Regular Expression” (by default “CHROM|HIGH|MODERATE”) to “CHROM|HIGH|MODERATE|SYNONYMOUS”. Then, launch the MOM workflow as usual.

### Including 5’UTR and 3’UTR variants

To include 5’UTR and 3’UTR variants, before launching the workflow, click on “Expand to full workflow form” (See Figure 1C) and scroll down to “Step 17: SnpEff eff”. Here, you will be able to change the parameters of SnpEff, the tool used to annotate the list of variants (VCF) called by Freebayes. At the option “Filter output”, select only “Do not show DOWNSTREAM changes”, “Do not show INTERGENIC changes”, “Do not show UPSTREAM changes (by default, “Do not show DOWNSTREAM changes”, “Do not show INTERGENIC changes”, “Do not show UPSTREAM changes”, “Do not show 5_PRIME_UTR or 3_PRIME_UTR changes” are selected). Then, scroll down to “Step 20: Search in text files”. At this stage, we use a regular expression to filter out all variants with no HIGH or MODERATE effect, including synonymous variants (based on SnpEff variant characterization - <http://pcingola.github.io/SnpEff/se_inputoutput/>). Change “Regular Expression” (by default “CHROM|HIGH|MODERATE”) to “CHROM|HIGH|MODERATE|UTR”. Then, launch the MOM workflow as usual.

### Including heterozygous variants

To include heterozygous variants, before launching the workflow, click on “Expand to full workflow form” (See Figure 1C) and scroll down to “Step 15: Variant calling (Freebayes) - Variant calling with Freebayes reporting: SNVs and InDels”. Here, you will be able to change the parameters of Freebayes, the tool used to detect the variants. At the option “Require at least this fraction of observations supporting an alternate allele within a single individual in the order to evaluate the position”, enter 0.4 instead of 0.9. Then, launch the MOM workflow as usual.

### Including LOW variants

To include LOW variants, before launching the workflow, click on “Expand to full workflow form” (See Figure 1C) and scroll down to “Step 20: Search in text files”. At this stage, we use a regular expression to filter out all variants with no HIGH or MODERATE effect, including synonymous variants (based on SnpEff variant characterization - <http://pcingola.github.io/SnpEff/se_inputoutput/>). Change “Regular Expression” (by default “CHROM|HIGH|MODERATE”) to “CHROM|HIGH|MODERATE|LOW”. Then, launch the MOM workflow as usual.

### Running several samples in parallel

MOM workflow on Galaxy offers the possibility to launch at once the analysis of several samples, in parallel. To set parameters for multiple samples analysis, upload all input files (FASTQs, reference genome, database, Orthology, and GO terms files) in one History. Upload the Workflow and click on the icon “Multiple datasets”
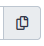
on the left of “Forward Fastq File (R1)” and “Reverse Fastq File (R2)” (see Figure 1 and Supplementary Figure S2). This will list all FASTQ files available in the current History. For “Forward Fastq File (R1), select all the forward FASTQ files (R1) by pressing “Ctrl” on the keyboard. Repeat that selection for “Reverse Fastq File (R2)” by selecting all R2 FASTQ files. Because MOM does not propagate the Sample ID up to the final CVL, we recommend changing the “Workflow Run Settings” (icon
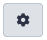
on the left of “Run Workflow) to enable the creation of a History per sample by selecting the option “Send results to a new history” (see Supplementary Figure S2). The analyses of each sample will be then conducted on different History, facilitating handling the datasets, and avoiding confusion between samples. Then, click on “Run Workflow”
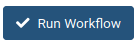
. The newly created History will be available in the list of your History. You can see the list by clicking on “Switch History”
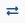
at the top of the History panel (left panel).

### Downloading and using it on your own galaxy server

Downloading and using it on your Galaxy server: For most cases Galaxy main (usegalaxy.org) or numbers of regional and local Galaxy server (<https://galaxyproject.org/use/>) is enough to run the analysis. But it is worth noting that it is possible to download the Galaxy platform and run it on a personal or private server.

## Building the Exclusion List with MOM workflow and Build_Exclusion_List workflow

### Selecting the right samples to build an Exclusion List

At a minimum, an Exclusion List should include variants called in reference strains such as N2 and CB4856 (Hawaiian) strains. Such Exclusion List is available at https://github.com/MTG-Lab/MOM. In addition, we recommend including all strains produced during the genetic screening.

### Step 1: Gathering VCFs

The first step consists in calling variants for all samples to be included in the Exclusion List. The expected format is VCF files. Optionally, the user could decide to use the MOM workflow to obtain the raw VCF file, including all homozygous variants called by Freebayes (file called “variantcalling_freebayes.vcf”). The file can be downloaded from the Galaxy History.

### Step 2: Uploading the Build_Exclusion_List workflow

The Build_Exclusion_list workflow is available at <https://usegalaxy.org/u/tmaroilley/w/buildexclusionlistmomworkflowv1>. Alternatively, in “Shared Data (top menu), in search workflow, type “exclusion” and the Build_Exclusion_List_MOM_workflow_v1 workflow will appear in the list of available workflows.

### Step 3: Uploading the VCFs

In a Galaxy history, gather (upload or copy) all VCFs to be included in the Exclusion List. Use “Upload Data” at the top of the Tool Shed.

Step 4: Transforming the VCFs into a collection dataset

The VCFs need to be transformed into a collection dataset. Once the VCFs are uploaded in the History, click on the checked box at the left top corner of the History. Then click of “Select All”. Expand the menu by clicking on “All selected” and choosing “Build Dataset List”. In the pop-up window, give a name to the Data Collection (e.g., “VCFs”) and click on “Create collection”. This new item in the History will be the input to the *Build_Exclusion_List* workflow. Supplementary Figure 4 shows screenshots of each step.

### Step 5: Running the Build_Exclusion_List workflow

The *Build_Exclusion_List* has only one input: the VCF Data Collection. Once selected, click on “Run Workflow”.

### Outputs

Once run, the *Build_Exclusion_List* workflow will create two outputs. The “*Exclusion_List_all*” is a text file resulting from the concatenation of all variants found in the different VCFs. This Exclusion List can be used if none of the samples included in the Exclusion List is the sample intended to be analysed. The “*Exclusion_List_only_Duplicates*” is a tsv file listing only variants find in at least two samples (duplicates). This should be used if the sample of interest has been included in the *Exclusion_List*, to avoid filtering out the modifier variant that should be unique to that strain.

## Analysis of the Joseph et al. (2018) dataset

Supplementary Table S1: Variants called and filtered by MOM in the Joseph et al. dataset.

| **Strain** | **Confirmed modifier (ce10)** | **Confirmed modifier (ce11)** | **Parame-ters** | **Variants** | **HIGH** | **MODE-RATE** | **LOW** | **CVL (only N2 and CB4856)** | **CVL** | **Confirmed modifier** |
| --- | --- | --- | --- | --- | --- | --- | --- | --- | --- | --- |
| WY1217 Sup | X:17,191,744-5bp-DEL | X:17,191,818 | Default | 1737 | 44 | 97 | 44 | 114 (88;26) | 3 (2;1) | YES |
| WY1217 Non sup | - | - | Default | 1754 | 42 | 100 | 47 | 114 (89;25) | - | - |
| WY1210 Sup | - | - | Default | 1795 | 49 | 111 | 54 | 134 (106;28) | - | - |
| WY1210 Non Sup | - | - | Default | 1808 | 48 | 105 | 49 | 126 (95;31) | - | - |
| WY1211 Sup | III:5,452,501-G-A | III:5,452,540 | Default | 1676 | 45 | 111 | 47 | 130 (102;28) | 6(6;0) | YES |
| WY1211 Non Sup | - | - | Default | 1755 | 43 | 105 | 47 | 121 (95;26) | - | - |
| WY1208 Sup | X:13,692,151-G-A | X:13,692,221 | Default | 1714 | 49 | 99 | 51 | 119 (90;29) | 8(7;1) | YES |
| WY1208 Non Sup | - | - | Default | 1709 | 45 | 96 | 52 | 114 (86;28) | - | - |
| WY1209 Sup | II:1,320,072-C-T | II:1,320,065 | Default | 1813 | 49 | 109 | 52 | 131 (100;31) |  | YES |
| WY1209 Non Sup | - | - | Default | 1706 | 42 | 100 | 48 | 116 (91;25) | - | - |
