# Supplementary figures and images for: Model Organism Modifier (MOM): a user-friendly Galaxy workflow to detect modifiers from genome sequencing data using Caenorhabditis elegans
Source: G3 (Bethesda). 2023 Aug 16;13(11):jkad184. doi: 10.1093/g3journal/jkad184 (PMC10627290; doi:10.1093/g3journal/jkad184)

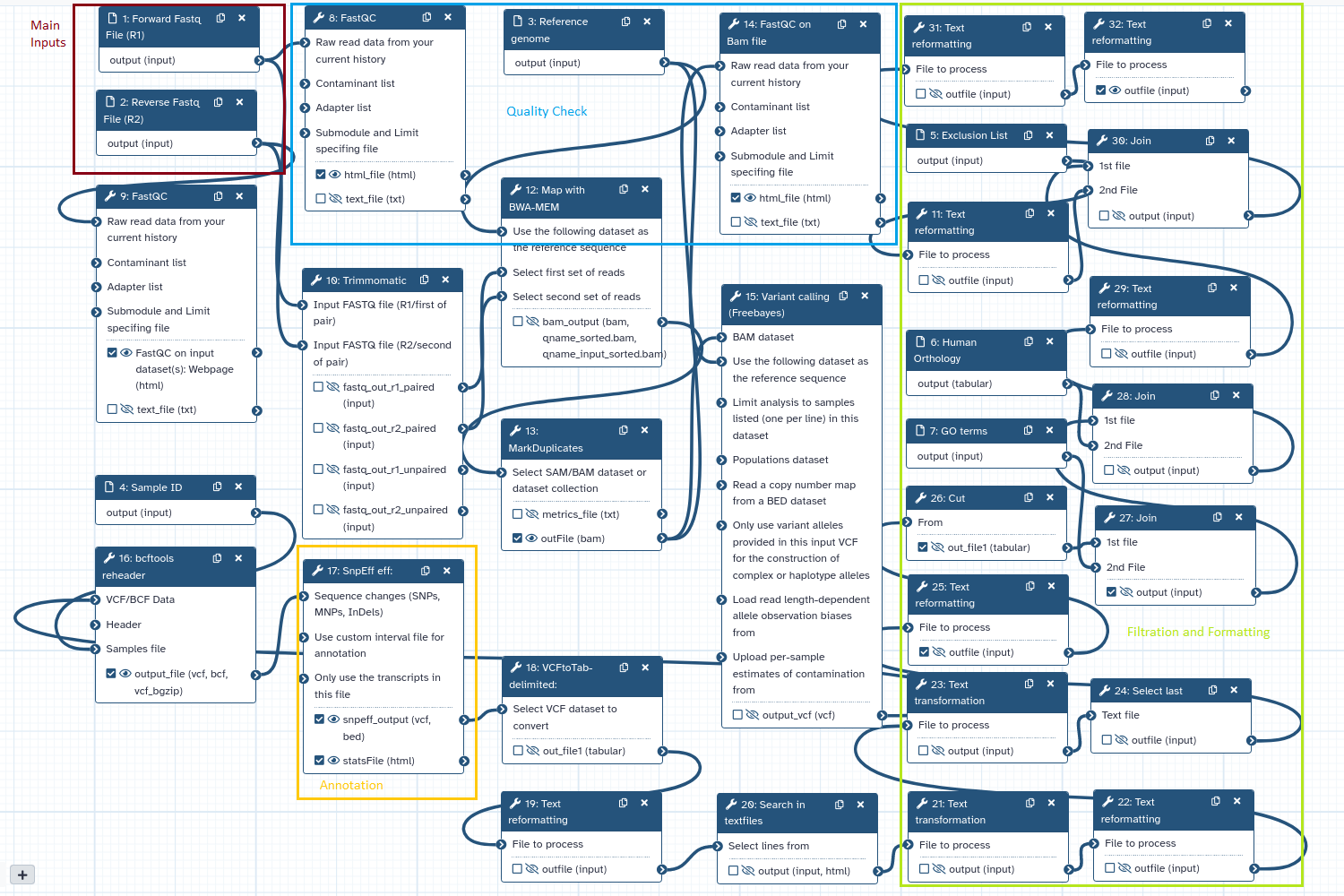

Supplement: jkad184_Supplementary_Data [file jkad184_supplementary_data.zip › Figure_S1_G3-2023-404449.tif]

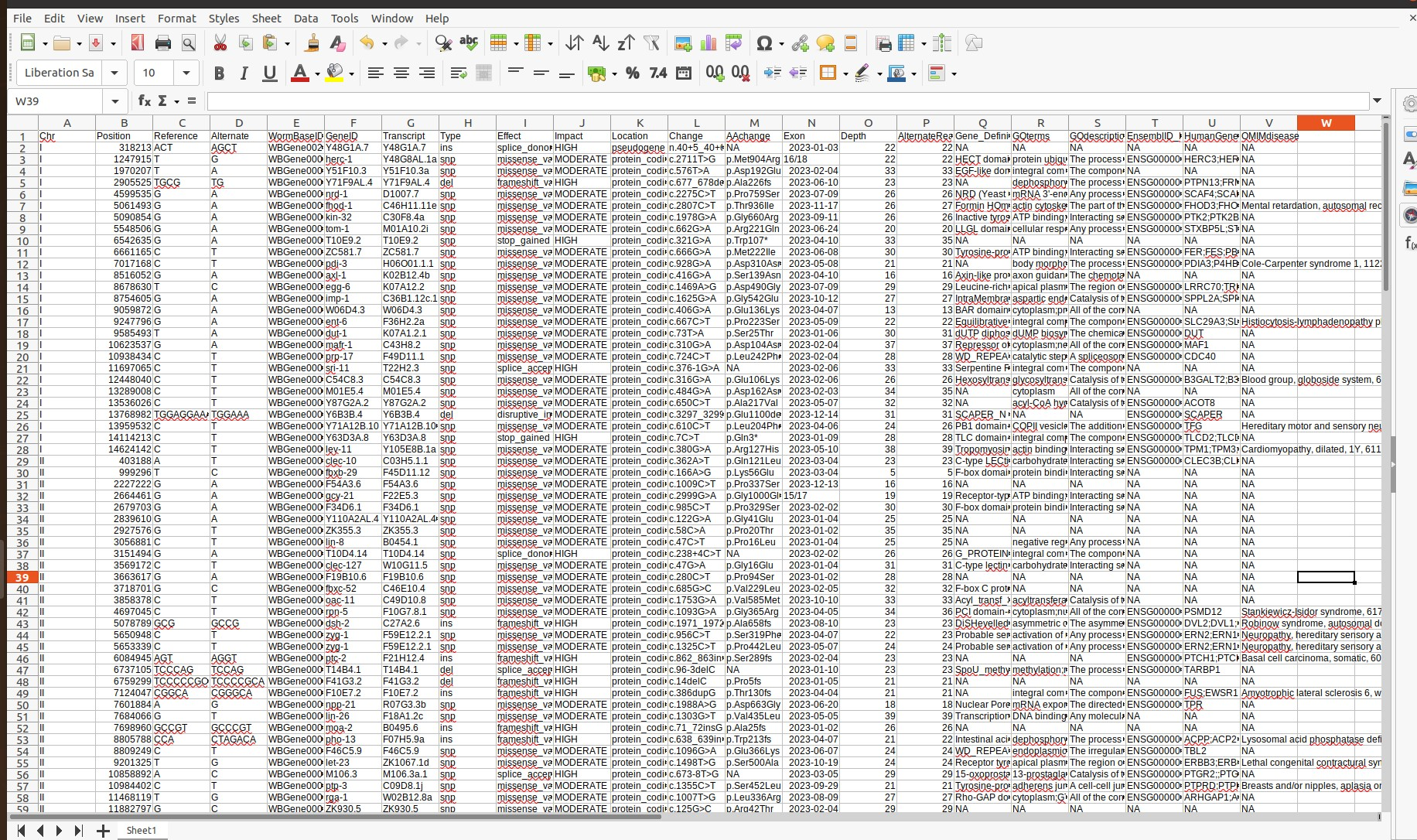

Supplement: jkad184_Supplementary_Data [file jkad184_supplementary_data.zip › Figure_S2_G3-2023-404449.tif]

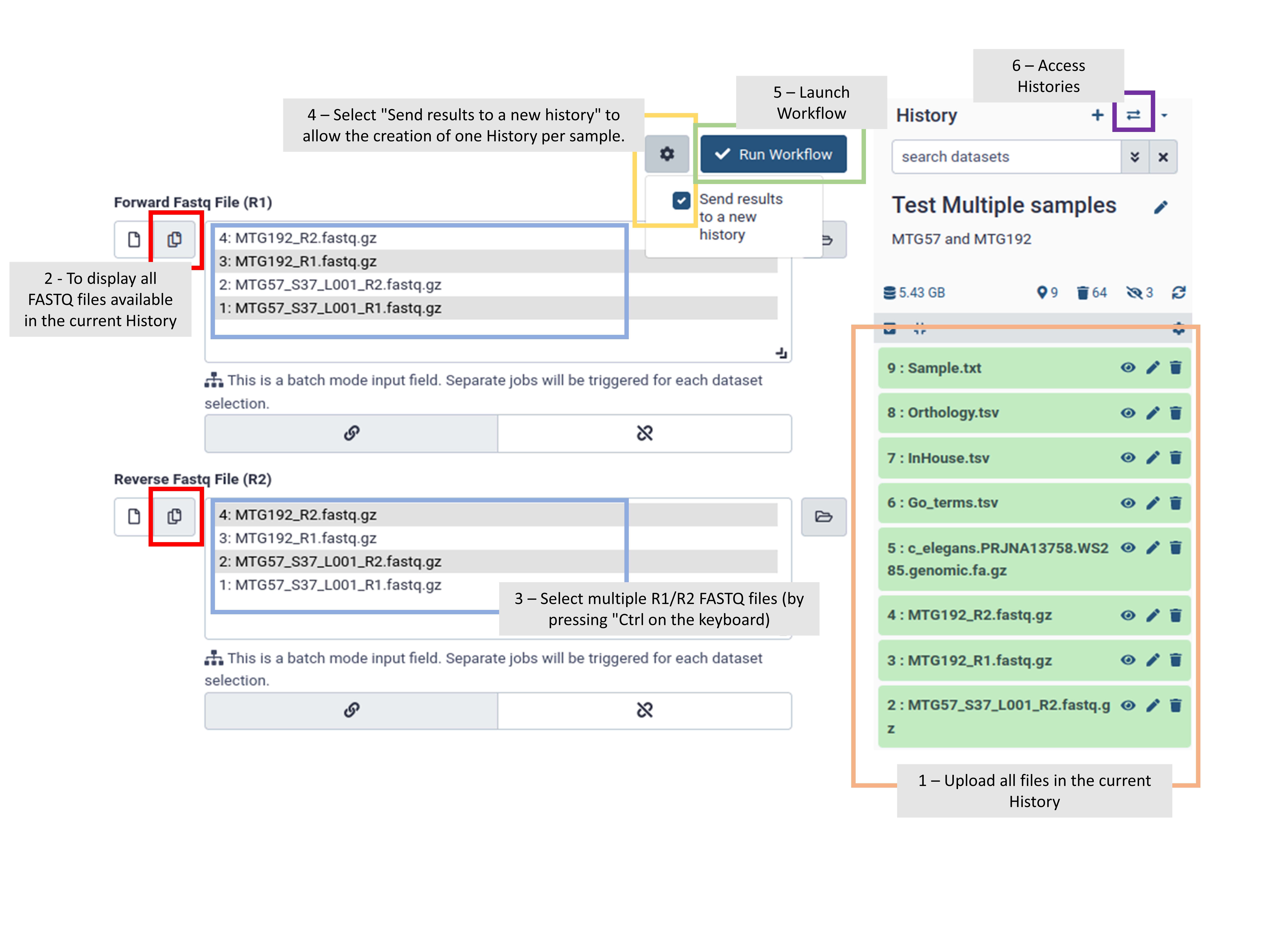

Supplement: jkad184_Supplementary_Data [file jkad184_supplementary_data.zip › Figure_S3_G3-2023-404449.tif]

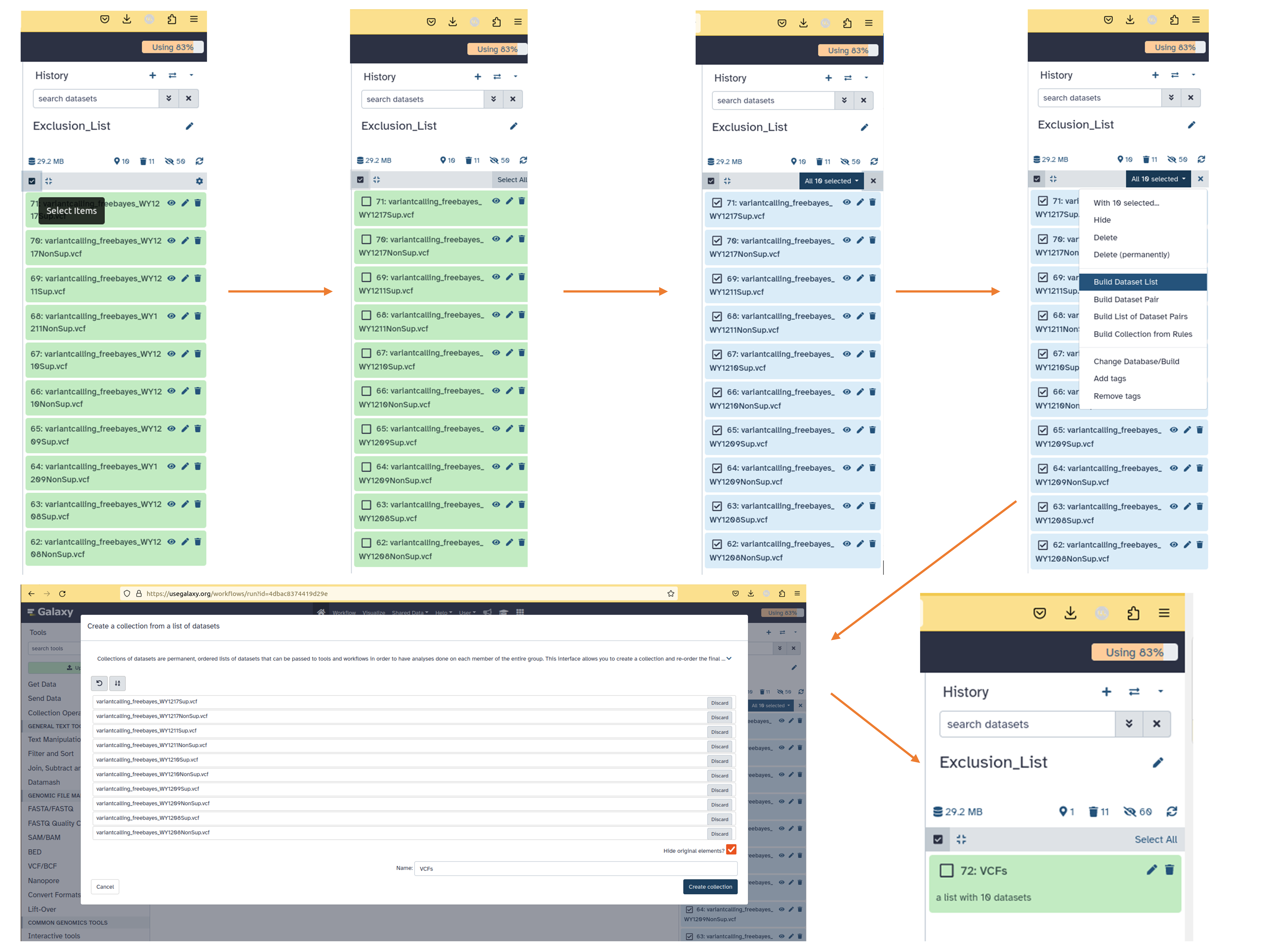

Supplement: jkad184_Supplementary_Data [file jkad184_supplementary_data.zip › Figure_S4_G3-2023-404449.tif]
